# Heart Rate Variability Indices as Possible Biomarkers for the Severity of Post-traumatic Stress Disorder Following Pregnancy Loss

**DOI:** 10.3389/fpsyt.2021.700920

**Published:** 2022-01-04

**Authors:** Cláudia de Faria Cardoso, Natalia Tiemi Ohe, Yazan Bader, Nariman Afify, Zahrah Al-Homedi, Salma Malalla Alwedami, Siobhán O'Sullivan, Luciana Aparecida Campos, Ovidiu Constantin Baltatu

**Affiliations:** ^1^Center of Innovation, Technology and Education (CITE), Anhembi Morumbi University, Sao Jose dos Campos, Brazil; ^2^Emory University, Atlanta, GA, United States; ^3^College of Medicine and Health Sciences, Khalifa University, Abu Dhabi, United Arab Emirates

**Keywords:** pregnancy loss experience, biomarkers, post-traumatic stress disorder, autonomic nervous system, heart rate variability

## Abstract

**Background:** Psychological distress, such as posttraumatic stress disorder (PTSD), is commonly evaluated using subjective questionnaires, a method prone to self-report bias. The study's working hypothesis was that levels of autonomic dysfunction determined by heart rate variability (HRV) measures are associated with the severity of PTSD in women following pregnancy loss.

**Methods:** This was an observational prospective cohort study with 53 patients enrolled. The DSM-5 (Diagnostic and Statistical Manual of Mental Disorders) PTSD scale (PCL-5) was used to assess the severity of PTSD in women after pregnancy loss. The cardiac autonomic function was assessed using HRV measurements during a deep breathing test using an HRV scanner system with wireless ECG enabling real-time data analysis and visualization. HRV measures were: standard deviation (SD) of normal R-R wave intervals [SDNN, ms], square root of the mean of the sum of the squares of differences between adjacent normal R wave intervals [RMSSD, ms], and the number of all R-R intervals in which the change in consecutive normal sinus intervals exceeds 50 milliseconds divided by the total number of R-R intervals measured [pNN50 = (NN50/n-1)^*^100%] [pNN50%].

**Results:** The PCL-5 scores had a statistically significant association with HRV indices (SDNN; RMSSD, and pNN50%). Patients with PTSD had similar mean heart rate values as compared to patients without PTSD (PCL-5), but significantly higher SDNN [median[IQR, interquartile range]: 90.1 (69.1–112.1) vs. 52.5 (36.8–65.6)], RMSSD [59.4 (37.5–74.9) vs. 31.9 (19.3 – 44.0)], and PNN50% values [25.7 (16.4–37.7) vs. 10.6 (1.5–21.9)]. The SDNN of the deep breathing test HRV was effective at distinguishing between patients with PTSD and those without, with an AUC = 0.83 +/− 0.06 (95 % CI 0.94, *p* = 0.0001) of the ROC model.

**Conclusions:** In this study, HRV indices as biomarkers of cardiac dysautonomia were found to be significantly related to the severity of PTSD symptoms in women after pregnancy loss.

## Introduction

Untimely pregnancy loss is an event that causes the mother to bear the burden of trauma that stretches well beyond the triggering incident. The extreme physiological and psychological symptoms suffered by those affected are grossly understudied (1). Previous research on the topic suggests a substantially elevated risk of depression and anxiety, but only minor evidence referring to posttraumatic stress disorder symptoms [PTSD; (2)]. PTSD after pregnancy loss is a type of trauma- and stressor-related disorder that is often misdiagnosed (3, 4).

Psychological stress such as PTSD is traditionally assessed by subjective reports, a method that is susceptible to self-report bias. The implementation of standardized and validated physiological and/or biological predictors of psychological stress would be a desirable solution to this problem (5). The rationale behind this study is based on the subjective nature of PTSD diagnosis. As elaborated upon in the DSM-5 (Diagnostic and Statistical Manual of Mental Disorders), many of the required criteria to diagnose someone with PTSD are prone to highly variable evaluation between clinicians (6). For instance, what is considered a “significant hindrance to daily normal function” is largely left to the subjective determination of the clinicians and the afflicted. In cases such as this, factors such as life responsibilities, working hours, and family dynamics all play a large role in one's perception of hindrance in their everyday life. Thus, the introduction of a more objective means of diagnosis, founded upon psychophysiological metrics, would allow for more reliable and accurate diagnosis of PTSD.

While prior literature suggests that PTSD is associated with autonomic nervous system (ANS) dysfunction, as documented by a recent meta-analysis (7), little research has been done into the possible autonomic dysfunction that may arise as a consequence of said PTSD among those who have lost a child during pregnancy.

The analysis of heart rate variability (HRV) in conjunction with the development of new algorithms is frequently used to detect changes in autonomic function that have predictive value in diseases (8). Widely-used metrics for HRV are time-domain, frequency-domain, and non-linear algorithms to assess changes of autonomic cardiac sympathetic and parasympathetic tone, or balance between parasympathetic and sympathetic modulation in health and diseases (9). Shaffer and Ginsberg (10) recently published an overview of heart rate variability metrics and norms.

The purpose of this study was to investigate cardiac dysautonomia as it relates to the severity of PTSD in women who have undergone pregnancy loss. For the scope of this study, cardiac dysautonomia refers to abnormal levels of heart rate variability (HRV). HRV is defined as the level of variability between individual heartbeats, and is often an indicator of the previously acquired stress response (7). The working hypothesis, as it pertains to this aim, is the expectation of a correlation between autonomic dysfunction and severity of PTSD symptoms.

## Methods

### Study Design and Setting

The study was carried out in accordance with the resolution 466/2012 and 340/2004 of the National Health Council (Ministry of Health) for research on human subjects, and with international medical ethics guidelines (Geneva Declaration, International Code of Medical Ethics, 1948, amend 1983). The study protocol was approved by the Ethics Committee of Anhembi Morumbi University (CAAE 13494719.7.0000.5492). Informed consent was obtained from each participant for the study.

This was a prospective cohort study that followed STROBE guidelines for reporting observational studies (11). This study recruited women who experienced perinatal loss and enrolled as patients and referred to the Department of Clinical Psychology at the Antenatal/Maternity Clinic at Hospital São Francisco de Assis, Jacareí, Brazil between January 2019 and June 2020.

One hundred and sixty-five women were invited to participate in the study. Women aged 18 to 47 years old with a history of at least one perinatal failure and no prior neurological or mental disorder were included in the study. A structured clinical interview was used to screen for present or past mental disorder in potential participants (12). The timeframe from the gestational process (at any time) until the first month of the baby's life was considered perinatal in this research. In total, 115 women were excluded, comprising 4 pregnant women, 54 who declined to attend the interview site, did not want to participate, or stopped participating after being scheduled, 46 who did not respond to the invitation, 10 who had out-of-date registers, and 1 woman who had an alcohol and/or illegal drug or dependency problem. The study recruited a cohort of 53 women, of which 20 women were recruited during hospitalization, 24 women from the institution's registry of medically required abortions and neonatal deaths, and 9 women by random demand.

### Diagnosis of Post-traumatic Stress Disorder (PTSD)

We used the Post-Traumatic Stress Examination List 5 (PCL-5) adapted to the Brazilian context (13). The operational definition for PTSD was based on the criteria as is presented in the Posttraumatic Stress Disorder Checklist (PCL-5) of the Diagnostic and Statistical Manual of Mental Disorders (DSM−5), which calls for the presence of at least one non-substance induced instance of each of the following categories that persists for at least a month: stressor, intrusion symptom, avoidance, negative affect or cognition (two symptoms), abnormal arousal/sensitivity, and significant hindrance to daily normal function (6). The PCL-5 contains 20 items, the answers to which are given along a 5-point Likert-type intensity scale, ranging from zero (nothing) to four (extremely). The cutoff point of 36 was considered predicting a PTSD diagnosis (13).

### Quantitative Autonomic Testing

#### Deep Breathing Test

Quantitative testing of the cardiac autonomic function was performed during deep breathing test, as previously described (14).

The heart rate response to deep breathing test has been considered as one of the most reliable of the cardiovascular tests of autonomic function (15, 16). The study of HRV during deep breathing test (or deep metronomic breathing; (17) is actually used to investigate cardiovascular autonomic function in a variety of pathologies. Ziemssen and Siepmann outlined the physiology, implementation, and assessment of HRV deep breathing in a recent review on the investigation of the cardiovascular and sudomotor autonomic nervous system (17).

**Figure 1:**
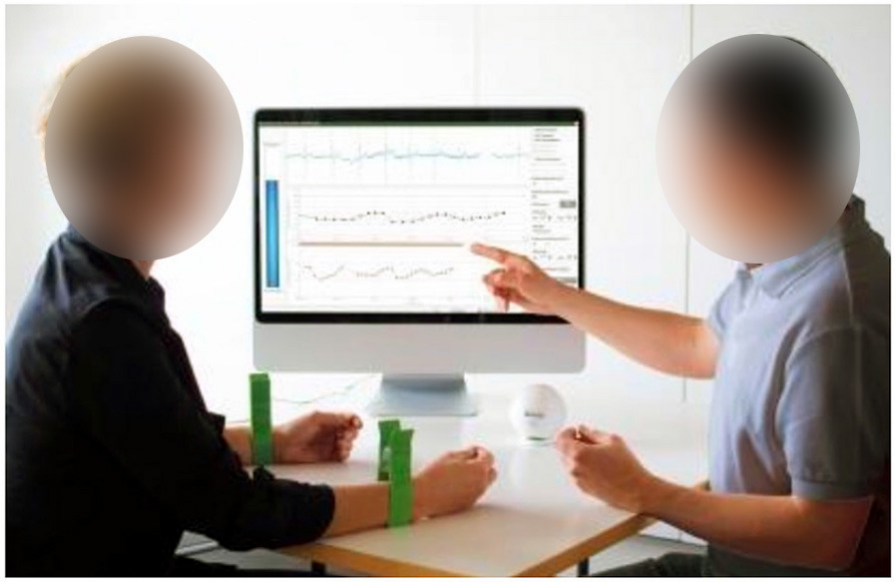
Evaluation of HRV with Deep Breathing Test by HRV Scanner. The patient, who is wearing wrist ECG electrodes, and the investigator are facing the computer screen; a bar instructing the patient on how to breathe is placed on the left side of the measurement window. The incoming biosignals, as well as the progression chart and measurement overview, are displayed in the center; HRV parameters are set in the configuration window on the right (representative picture–source: Mega HRV-Scanner Flier 02__2013 E DB-v.1.1).

The HRV deep breathing test was measured in real-time and online with Faros wireless ECG monitor and HRV-scanner software (Mega Electronics, Finland) that uses a respiration pacer while measuring and analyzing the variability in the heart rate in response to deep breathing (Figure 1). The subjects were instructed to follow the respiration pacer bar, and to breathe as deeply as possible. The tests were performed with subjects in sitting position over six respiratory cycles and a respiratory rate of six breaths per min. Cardiac autonomic function was evaluated measuring the time-domain HRV parameters: standard deviation (SD) of normal R-R wave intervals [SDNN, ms] (18, 19), square root of the mean of the sum of the squares of differences between adjacent normal R wave intervals [RMSSD, ms] (20), and the number of all NN intervals in which the change in consecutive normal sinus intervals exceeds 50 milliseconds divided by the total number of NN intervals measured [pNN50 = (NN50/n-1)^*^100%] [pNN50%] (10).

#### COMPASS-31 (Composite Autonomic Symptom Score)

General autonomic dysfunction may be evaluated with the Composite Autonomic Symptom Score 31 (COMPASS 31) (21). The Compass 31 Questionnaire has a total of 31 questions across 6 domains: orthostatic intolerance, vasomotor, secretomotor, gastrointestinal, bladder, and pupilomotor. The Portuguese version of Compass 31 questionnaire was utilized in this study (22). In short, the higher the score, the more severe the autonomic dysfunction.

### Study Protocol

After establishing rapport, the patient was presented with the research project for 10-15 min, with the required written consent. Following that, an unstructured interview lasting ~60 min took place. The patient spoke spontaneously throughout this section of the interview about her gestation history and the impact of loss on her life and family. Following the interview, the patient was instructed to spend ~20–30 min completing the questionnaires PCL-5 and Compass-31. A 1 min deep breathing test was administered at the conclusion of the visit. To obtain reliable results, the deep breathing test was always performed in accordance with the study protocol and in the same consulting room under controlled conditions.

### Statistical Analysis

Data were tested for normality distribution using D'Agostino-Pearson normality test (23) and Kolmogorov-Smirnov test with Dallal-Wilkinson-Lilliefors' *P*-value (24). Differences between study groups were assessed with Mann-Whitney non-parametric test. Spearman's correlation coefficient r was used to measure a relationship between DSM-5 scales and HRV indices or COMPASS scores. The correlation intensity was rated as negligible (0.30), low (0.30–0.50), moderate (0.51–0.70), high (0.71–0.90), and very high (>0.90) (25). Diagnostic utility was evaluated using the area under the receiver-operating characteristic (ROC). To measure the diagnostic accuracy, the following indices were used: area under the curve (AUC) with standard error (SE) and its binomial exact 95% confidence interval, Youden's J index, sensitivity (Sn), and specificity (Sp). All statistical analyses were carried out using GraphPad Prism version 8.1.2 for Mac OS X (GraphPad Software, La Jolla California USA, www.graphpad.com). Differences were considered significant when the probability of a Type I error was lower than 5% (p < 0.05).

## Results

### Patient Characteristics and Outcomes

In the recruited cohort of 53 participants, 25 were diagnosed with pregnancy loss-induced PTSD and 28 were not based on self-reported PCL-5 scores. Patients were on average 33 years old [25–39] [median(IQR)]. Pregnancy loss intervals ranged from less than 40 days to more than 6 months: <30 days (23 patients), 31–90 days (10 patients), 3–5 months (9 patients), and >6 months (11 patients). Gestational age ranged from 4–42 weeks: 4–14 weeks (36 patients), 15–22 weeks (8 patients), 23–28 weeks (4 patients), 29–35 weeks (1 patient), and 36–42 weeks (4 patients). There were no significant differences on the investigated outcomes between these study subgroups because the study was not designed or powered sufficiently to detect such differences. Descriptive statistics for heart rate variability indexes from deep breathing test are presented in Table 1 as median and interquartile range values.

**Table 1:** Characteristics of heart rate variability indexes from deep breathing test [Median (interquartile range, IQR)].

**HRV Index**	**Median [IQR]**
Mean heart rate	80.70 [74.33–88.66]
SDNN (ms)	66.47 [46.92–91.03]
RMSSD (ms)	39.91 [28.20–59.69]
PNN50%	17.44 [7.20–30.33]

### Associations Between Heart Rate Variability Indices or COMPASS Scores With PCL-5 Scores

**Figure 2:**
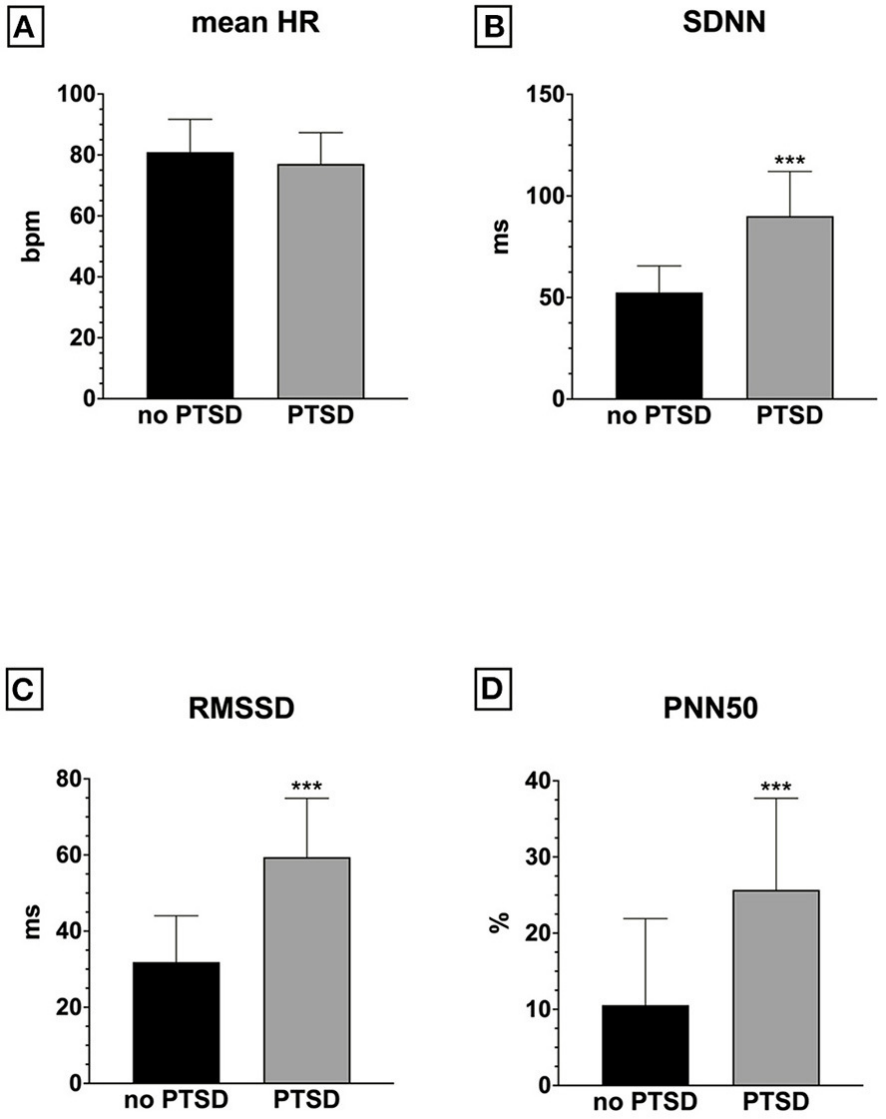
Heart rate variability indices in PTSD versus no PTSD: **(A)** mean HR; **(B)** SDNN; **(C)** RMSSD; **(D)** PNN50; ***p < 0.001.

Patients with PTSD had similar mean heart rate values (Figure 2A) as compared to patients without PTSD (PCL-5), but significantly higher SDNN (Figure 2B), RMSSD (Figure 2C), and PNN50 values (Figure 2D).

**Figure 3:**
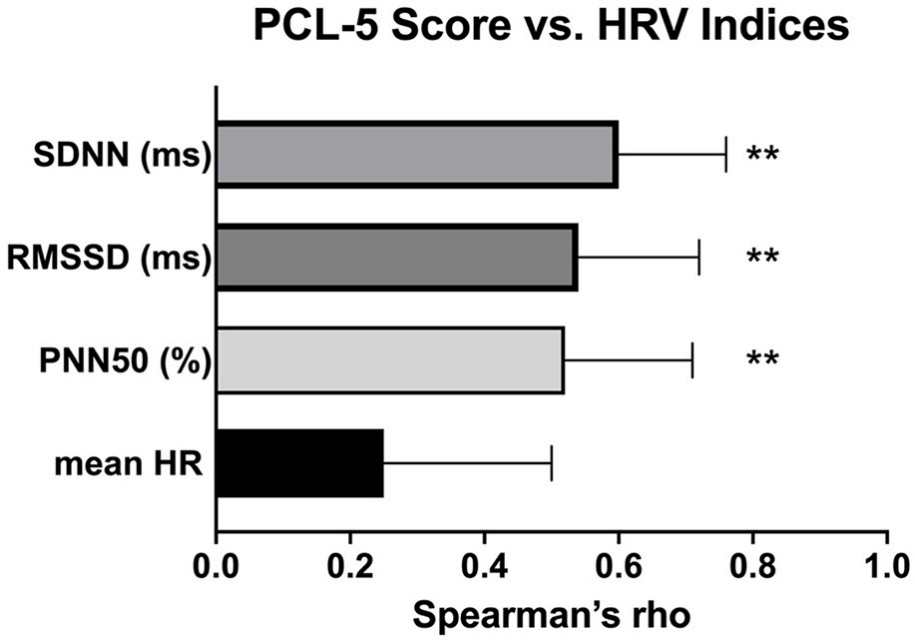
Correlations between HRV inidices and PCL-5 scores. **p < 0.01.

There were significant positive correlations between HRV indices and PCL-5 scores as indicated by a Spearman's rho (Figure 3). Among these HRV indices, SDNN had the strongest correlation with PCL-5 scores.

A significant positive association was found between the Bladder Disorder domain of the COMPASS-31 score and PCL-5 scores (Table 2). Also, there was a significantly positive but weak correlation between pupillomotor COMPASS-31 score and PCL-5 scores.

**Table 2:** Correlations of the PCL-5 scores with COMPASS scores.

	**PCL scale score vs. COMPASS Total**	**PCL scale score vs. COMPASS Bladder Disorder**	**PCL scale score vs. COMPASS Pupillomotor**	**PCL scale score vs. COMPASS Orthostatic Intolerance**	**PCL scale score vs. COMPASS Vasomotor**	**PCL scale score vs. COMPASS Secretomotor disorder**	**PCL scale score vs. COMPASS Gastrointestinal**
**Spearman r**							
r	0.30	0.40	0.31	0.29	0.19	0.23	0.07
95% confidence interval	0.03–0.53	0.14–0.61	0.03–0.54	0.007–0.52	−0.09–0.45	−0.05–0.47	−0.21–0.34
*P*-value (two-tailed)	0.0279	0.0029	0.025	0.0387	0.1657	0.103	0.605

### Prediction Value of the SDNN HRV Index for the Severity of PTSD

**Figure 4:**
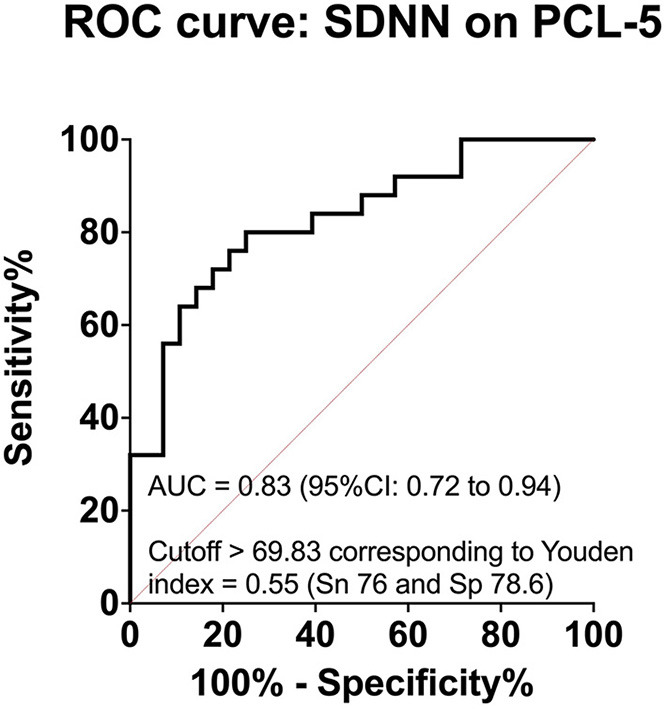
Prediction value of SDNN HRV index for the severity of PTSD.

With an AUC = 0.83 (*p* < 0.001) of the ROC model, the deep breathing test HRV's SDNN was good at distinguishing between patients with PTSD and those with no PTSD diagnosed with a cutoff point of 36 of PCL-5 scores (Figure 4). The cutoff value was > 69.8, corresponding to Youden index 0.55, with a sensitivity prediction of 76.0% and specificity 78.6%.

## Discussion

In this study, we found that women who had undergone pregnancy loss were at significantly greater risk of experiencing cardiac dysautonomia; the severity of this was found to correlate with that of PTSD and acute stress disorder as evaluated by DSM-5 PTSD scale (PCL-5 scale). These findings provide further evidence to support the notion that women who had undergone miscarriage were disproportionately affected by PTSD symptoms; from this, one could point to the need for improved diagnosis of trauma-, stressor-, and anxiety-related disorders in this category of women. Moreover, all patients perceived the HRV deep breathing test with the HRV scanner as an improved care with the visualization of objective measures.

These findings build upon those previous studies that uncovered signs toward PTSD as a consequence of pregnancy loss (2). Our study incorporated a physiological metric (HRV) which could be used as a quantitative biomarker of PTSD alongside self-reported evaluation. That this physiological measure's intensity correlates with the severity of PTSD symptoms is further evidence that PTSD may pose a threat to women postpartum.

Patients with PTSD are usually characterized as having an autonomic nervous system dysregulation, typically involving a hyperactive sympathetic tone, as observed in this study (26). HRV research offers information on how the autonomic nervous system (ANS) functions (8). HRV parameters determined using time-domain, frequency-domain, and non-linear methods have shown that PTSD has an effect on both the sympathetic and parasympathetic nervous systems (7). Previous research on PTSD has indicated that patients with PTSD may have lower HRV than controls, implying altered sympathetic and parasympathetic behavior (5). However, the results are inconsistent, possibly due to the different natures of trauma, the omission of potential PTSD subtypes, or possibly due to a remarked publication bias among the meta-analysis findings, as Pole and Campbell et al. noted (27, 28). Another possible explanation for the apparent discrepancy between these findings of increased HRV indices in PTSD and previous reports of decreased HRV in PTSD is that these studies examined different time periods of PTSD progression. The PTSD patient population studied so far is diverse, with a wide range of traumatic experiences and demographics. As a consequence, the standard magnitude of the disorder's effect on HRV cannot be assumed (29). In this study, we investigated how pregnancy loss affected physiological measures associated with PTSD, such as HRV indices. SDNN, which reflects parasympathetic and sympathetic modulation (18, 19), as well as RMSSD and PNN50, which reflect cardiac parasympathetic modulation (18, 30), were found to be able to discriminate between patients with pregnancy-related PTSD and those without PTSD. The PTSD-induced augmented sympathetic modulation observed in this study is consistent with previous research indicating that catecholaminergic system hyperactivation may be associated with PTSD symptoms (31). Such findings laid the groundwork for research into medications that suppress the catecholaminergic system as a treatment for PTSD symptoms. Prazosin, an alpha-1 adrenergic receptor antagonist, for example, was effective in treating PTSD symptoms (32, 33). In such clinical trials, HRV indices of autonomic dysfunction could be used as predictive pharmacological biomarkers to aid in the clinical development of drugs or non-pharmacological therapies while minimizing adverse effects in the individual PTSD patient (34).

The COMPASS-31 questionnaire is a validated outcome test that can be used to monitor autonomic symptoms and track treatment response. COMPASS-31 has been reported as a useful tool for the evaluation of autonomic dysfunction in various diseases (35). COMPASS-31 was recently used in two studies to examine autonomic symptoms in PTSD patients (36, 37). In this study, a significant positive association was shown between PTSD and the bladder and pupillometry indices on the COMPASS-31. The clinical significance of this finding must be interpreted in light of expected versus unexpected bladder issues, as these are common pregnancy and abortion complications (38). Further research with a larger cohort of patients suffering from pregnancy loss-induced PTSD could shed light on the autonomic dysfunction of specific domains, as COMPASS-31 may be used as a sensitive and convenient screening tool (39). Furthermore, as has been shown for type 2 diabetes, combining the COMPASS-31 and HRV indices could improve the diagnostic performance of autonomic dysfunction in PTSD (40).

### Limitations of the Study

There are several limitations that should be taken into consideration. First, since this is a cross-sectional analysis, it is difficult to draw predictive conclusions. Longitudinal studies are needed to investigate the evolution of the identified HRV indices alongside the related PTSD. The second drawback is selection bias, which is due to the existence of high-risk PTSD patients that are referred directly from the Antenatal/Maternity Clinic. As a result, prevalence-incidence bias (also known as Neyman bias) must be recognized since the sampling procedure resulted in fewer participants with mild disease in the study, which may lead to an error in the calculated relationship between an exposure and an outcome.

Further studies shall be designed to investigate the effects of relevant confounding variables on the associations between PTSD and cardiac dysautonomia, such as time from the traumatic event, demographics (age, sex, race/ethnicity), or traditional CVD risk factors (hypertension, diabetes, smoking, total cholesterol, and high density lipoprotein). While the study was successful in recruiting a cohort of 53 patients with possible PTSD over the course of 18 months after pregnancy loss, the finding's generalizability must be further investigated in different cohorts. Furthermore, the study's applicability to other clinical cases and other populations requires further investigation.

## Conclusion

In this study, cardiac dysautonomia was consistently associated with the severity of PTSD symptoms after pregnancy loss. It may be reasonable to conclude that further research could therefore establish HRV measures as an integrated diagnostic tool for cardiac dysautonomia associated with PTSD from different traumas and other stress disorders. Further studies of HRV measures on PTSD may provide insight into psychological recovery processes as well as serve as a guide for treatment correspondence.

## Data Availability Statement

The raw data supporting the conclusions of this article will be made available by the authors, without undue reservation.

## Ethics Statement

The study protocol was approved by the Ethics Committee of Anhembi Morumbi University (CAAE 13494719.7.0000.5492). Informed consent was obtained from each participant for the study. The patients/participants provided their written informed consent to participate in this study.

## Author Contributions

LC, OB, and CF: study conception and design. CF and NO: performed the study. CF, LC, SO'S, and OB: assays and data analysis. LC, OB, SO'S, CF, YB, NA, ZA-H, and SA: interpretation of the data, writing of the manuscript, and critical revision of the manuscript regarding the important intellectual content. All authors contributed to the article and approved the submitted version.

## Funding

This research was funded by the Center of Innovation, Technology and Education (CITE, 2018-005) and by Khalifa University of Science and Technology (Award No. FSU-2020-33) to OB. OB was supported by the National Council for Scientific and Technological Development (CNPq, 307760/2018-9). CF received an Anhembi Morumbi University-Laureate International Universities Master's scholarship.

## Conflict of Interest

The authors declare that the research was conducted in the absence of any commercial or financial relationships that could be construed as a potential conflict of interest.

## Publisher's Note

All claims expressed in this article are solely those of the authors and do not necessarily represent those of their affiliated organizations, or those of the publisher, the editors and the reviewers. Any product that may be evaluated in this article, or claim that may be made by its manufacturer, is not guaranteed or endorsed by the publisher.

## Abbreviations

PTSD: Posttraumatic Stress Disorder
DSM-5: Diagnostic and Statistical Manual of Mental Disorders, 5th Edition
PCL-5: Posttraumatic Stress Disorder Checklist for DSM-5
HRV: heart rate variability
ECG: electrocardiography
RR: interval between two heartbeats (R spikes in the QRS complex / ECG)
SDNN: standard deviation (SD) of normal R-R wave intervals
RMSSD: square root of the mean of the sum of the squares of differences between adjacent normal R wave intervals
pNN50%: the number of all R-R intervals in which the change in consecutive normal sinus intervals exceeds 50 milliseconds divided by the total number of R-R intervals measured [pNN50 = (NN50/n-1)^*^100%]
ANS: autonomic nervous system
COMPASS 31: Composite Autonomic Symptom Score 31
ROC curve: receiver operating characteristic curve.
