# Supplementary material for: Heart Rate Variability Indices as Possible Biomarkers for the Severity of Post-traumatic Stress Disorder Following Pregnancy Loss
Source: Front Psychiatry. 2022 Jan 4;12:700920. doi: 10.3389/fpsyt.2021.700920 (PMC8763675; doi:10.3389/fpsyt.2021.700920)
Supplement: Supplementary file 1 [file Data_Sheet_1.PDF]

HRV

HRV-Scanner -Software: Diagnostics and Biofeedback

A well-functioning neuro-vegetative regulation is the key to the health and the well-being of your patients/clients.

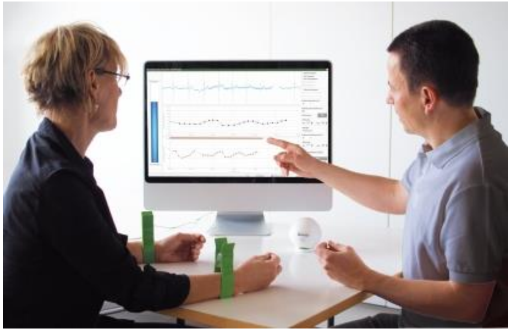

Whether HRV measurement (HRV-scan) or heart (HRV) bio feedback, the *BioSign HRV technology* is successfully applied by doctors, psychologists, therapists, coaches and management trainers.

The expertise provided in the HRV scanner is based on years of experience in the HRV diagnosis in various application areas. Basis for this are especially numerous scientific studies and publications on the heart rate variability.

- HRV biofeedback system (3D animated Biofeedback, Real time spectral analysis – online spectrum)
- HRV test lab (HRV standard tests, like e.g. RSA test [deep breathing test], Short term HRV, Ewing's - and Orthostasis test, long term measurement)
- HRV long term measurement 24 h (colour-coded spectral analysis) with synchronous display of the body position using the integrated 3D-accelerometer of eMotion HRV+3D (image below)

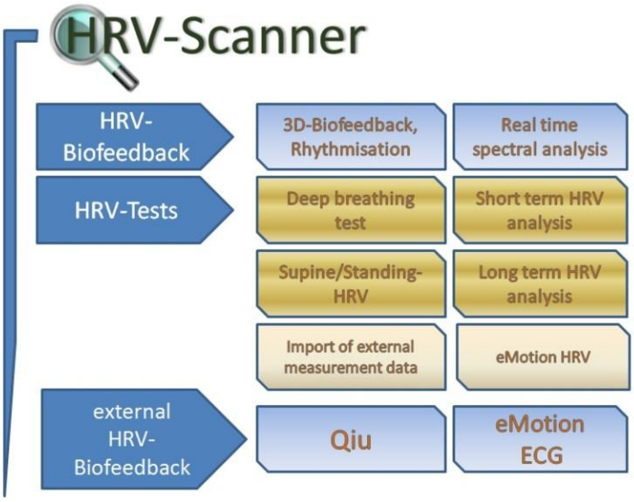

The HRV scanner software differs from other HRV analysis and biofeedback systems through the transparency, accountability and objectivity of the calculated and presented data and graphics.

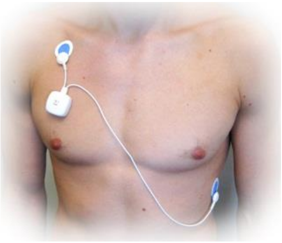

24h HRV + body position

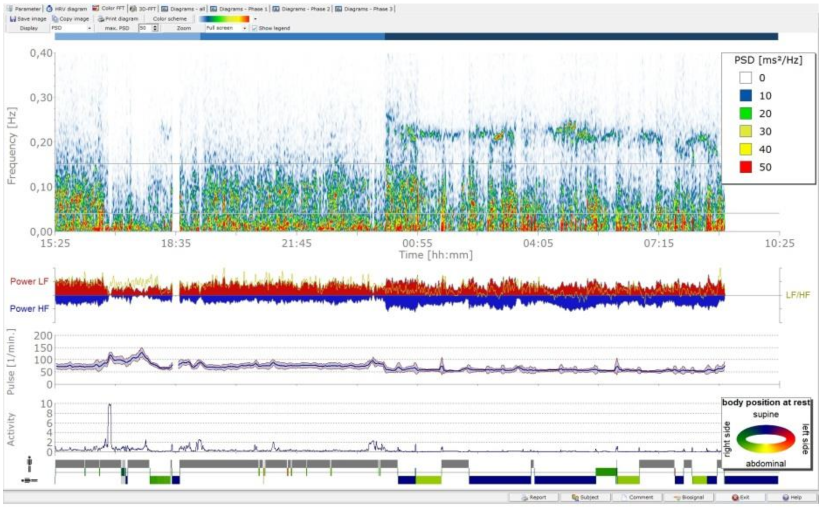

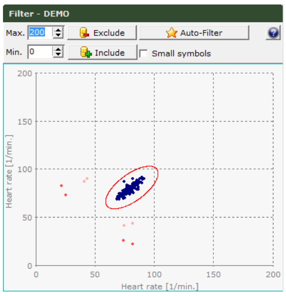

The analysis module provides extensive post-processing options in addition to the automated quality assessment.

Meaningful charts and illustrations, as well as many parameters (for the main, standard values are stored) allow a quick and meaningful interpretation of the test results. A comprehensive reporting system creates easy-to-understand reports to therapist and clinical trials.

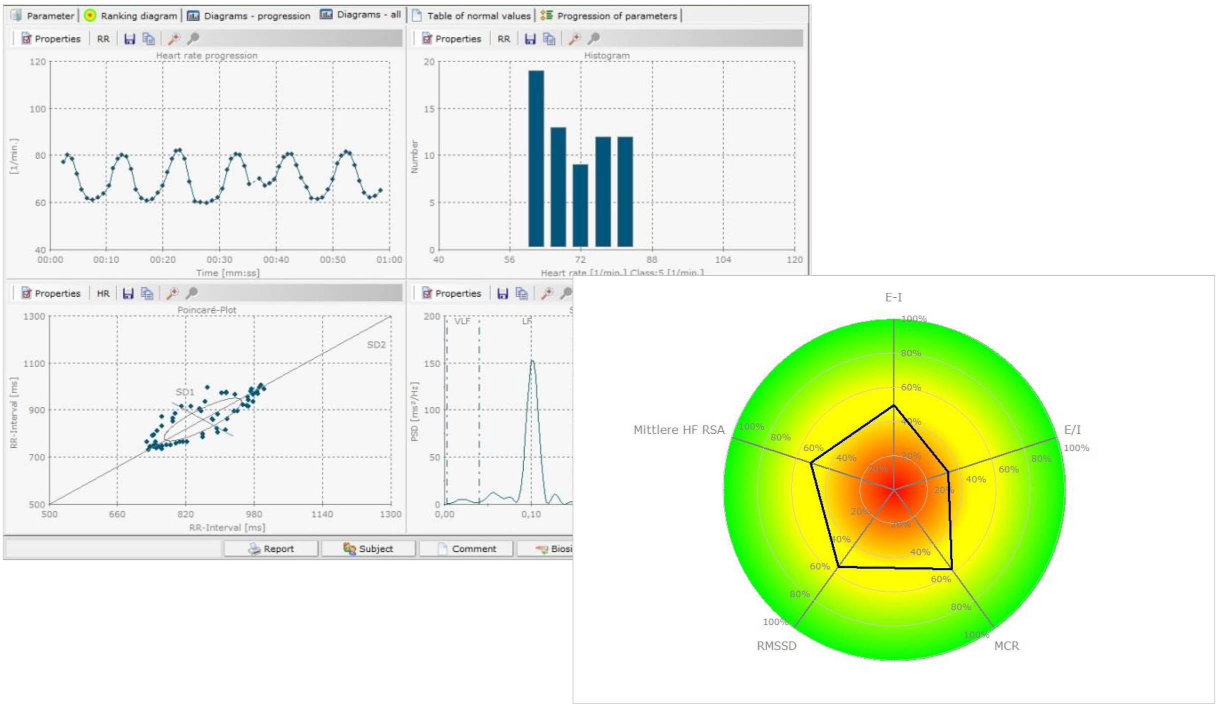

For users who want to work scientifically, there is a study module available, which allows the collection and processing of study data effectively and clearly in the HRV scanner. Export functions to interface external applications facilitate the work (e.g. statistics programs).

Table overview of the HRV scanner software with usable measurement modules and accessories:

**Application**	**Needed hardware**	**Order-No.** (Mega Electronics)
3D-HRV-Biofeedback	eMotion ECG (2 connectors) with arm clamps or surface electrodes	900750 (eMotion ECG)
Realtime spectral analysis		
RSA measurement		
Short-term HRV		900752 (Arm clamps)
Supine/Standing HRV		Ambu M-00-S
External HRV/Biofeedback	QIU ball	QIU
Long term HRV up to 5 days	eMotion HRV	900477
Long term HRV+3D: 24h HRV + actimeter + body position	eMotion HRV+3D	900675

For more details, you receive the current mega HRV price list on request.

Users of ***HRV Scanner*** in the USA please note:
Caution – For Research Use Only. Not for use in diagnostic procedures. Device may be used for non-clinical laboratory research
